# Etanercept-induced palisaded neutrophilic granulomatous dermatitis^☆^^☆☆^

**DOI:** 10.1016/j.abd.2020.06.005

**Published:** 2020-11-16

**Authors:** Masato Ishikawa, Toshiyuki Yamamoto

**Affiliations:** Fukushima Medical University, Fukushima, Japan

*Dear Editor,*

A 37-year-old female was referred to this hospital, complaining of fever and induration and swelling of the chest wall. She had rheumatoid arthritis and been treated with non-steroidal anti-inflammatory drugs and methotrexate for six years, as well as etanercept (25 mg per three weeks) for two years, prior to presentation. Physical examination revealed a reddish induration with tenderness and swelling of the left breast (Fig. 1). It was also accompanied by a number of tiny pustules. Laboratory data showed positive anti-nuclear antibody (1:160), elevated C-reactive protein (14.91 mg/dL), anti-SS-A antibodies (> 240 U/mL), and positive Schirmer’s test (left eye; 2 mm). The patient had not been diagnosed as having Sjögren’s syndrome until presentation at this hospital. A biopsy specimen showed basophilic degeneration of collagen fibers mixed with numerous nuclear debris in the upper dermis (Fig. 2). There was also mild interface dermatitis. Immunohistochemistry results revealed a number of CD68-positive palisaded histiocytes surrounding degenerated collagen fibers (Fig. 3). Although the patient was treated with antibiotics for cellulitis initially, these had little effect. Since the patient did not show any other obvious symptoms, several studies were performed in order to identify the source of fever. Examination of cerebrospinal fluid revealed that she had aseptic meningitis, which was considered as a part of the central nervous system involvement in autoimmune disease. The patient’s fever and induration improved with 50 mg of prednisolone. After recovery, etanercept therapy was stopped. Although the patient continued treatment with non-steroidal anti-inflammatory drugs and methotrexate, three months after stopping etanercept, she was free from recurrence.

**Figure 1:**
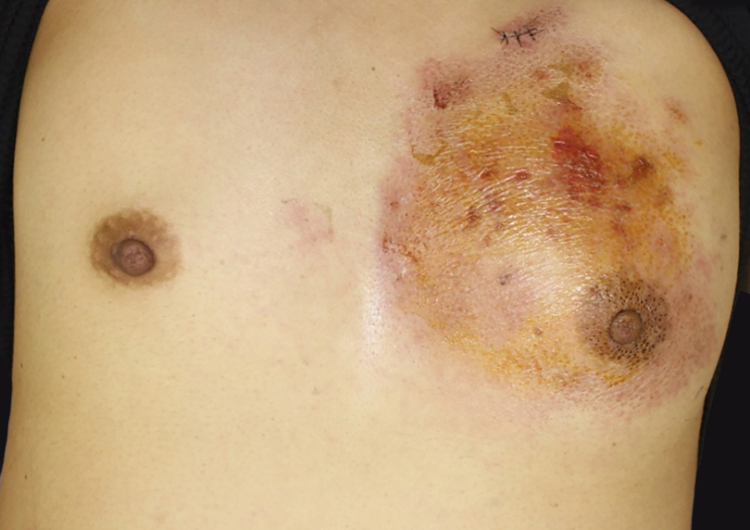
Clinical features of the trunk. A reddish induration with tenderness and swelling on the left breast.

**Figure 2:**
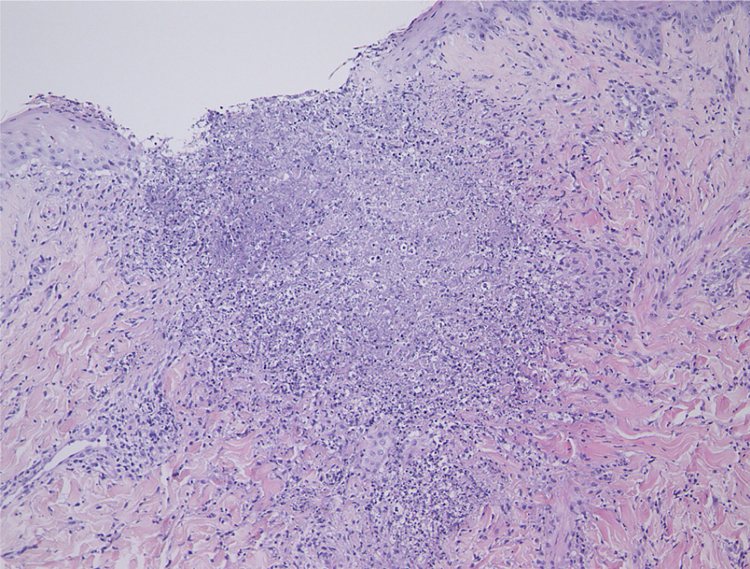
The histopathological specimen showed basophilic degeneration of collagen fiber, mixed with numerous nuclear debris in the upper dermis (Hematoxylin & eosin, ×100).

**Figure 3:**
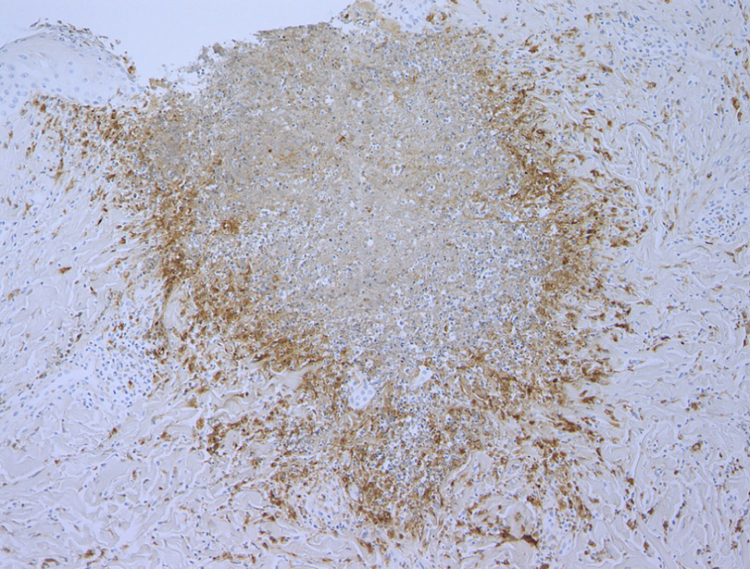
Palisaded histiocytes surrounding degenerated collagen fibers (CD68, ×100).

In the present case, infiltrative erythema occurred in a patient with rheumatoid arthritis and Sjögren’s syndrome two years after starting treatment with etanercept, and the eruption has not occurred since the etanercept treatment was stopped. Histopathological examination revealed characteristic findings of palisaded neutrophilic granulomatous dermatitis (PNGD) and interface dermatitis. Considering the clinical course and the histopathological features, PNGD could be induced by etanercept in the present case. PNGD is a pathological concept characterized by an inflammation pattern showing a palisading infiltration of histiocyte surrounding areas of necrobiosis, which occasionally occurs in patients with autoimmune diseases such as rheumatoid arthritis, Sjögren’s syndrome, and eosinophilic granulomatosis with polyangiitis.^1^ To date, there have only been few reported cases of PNGD occurring after treatment with etanercept, including the present case.^2^, ^3^, ^4^ In contrast to other TNF-α inhibitors, etanercept, a receptor fusion protein, is considered not to strongly suppress TNF-α activity and this may enable TNF-α to form granuloma. Otherwise, etanercept may modulate cytokines other than TNF, which cannot be done by other TNF inhibitors. In addition, etanercept can enhance T-cell production of interferon-gamma, which is a key player in granuloma formation.^5^ By contrast, there are reports that adalimumab and infliximab also induce sarcoid or sarcoid-like granulomas, and thus other mechanisms leading to granuloma induction may exist. Further studies are necessary to clarify the mechanisms of TNF inhibitors-induced granulomatous diseases.

## Financial support

None declared.

## Authors' contributions

Masato Ishikawa: Design and planning of the study; drafting and editing of the manuscript; collection, analysis, and interpretation of data; critical review of the literature.

Toshiyuki Yamamoto: Approval of the final version of the manuscript; design and planning of the study.

## Conflicts of interest

None declared.
